# *N*-Acetyl-2-hydroxy-*N*′-[methoxy(1-methylindol-2-yl)methyl]benzohydrazide

**DOI:** 10.1107/S1600536808026846

**Published:** 2008-08-23

**Authors:** Wagee A. Yehye, Noorsaadah Abdul Rahman, Azhar Ariffin, Seik Weng Ng

**Affiliations:** a Department of Chemistry, University of Malaya, 50603 Kuala Lumpur, Malaysia

## Abstract

In the crystal structure of the title Schiff-base, C_20_H_21_N_3_O_4_, the amino group forms an N—H⋯O hydrogen bond to the acetyl group of an adjacent mol­ecule, forming a zigzag chain. The 2-hydr­oxy group is inter­nally hydrogen bonded to the amido group though an O—H⋯O hydrogen bond.

## Related literature

For medicinal uses of the precursor Schiff base, see: Jin *et al.* (2006 ▶); Joshi *et al.* (2008 ▶); Szczepankiewicz *et al.* (2001 ▶).
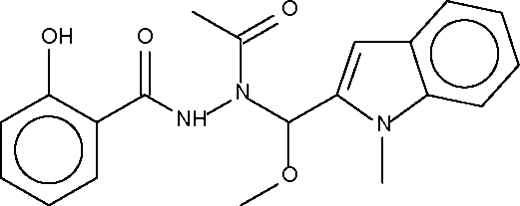


## Experimental

### 

#### Crystal data

C_20_H_21_N_3_O_4_
*M* *_r_* = 367.40
Monoclinic, 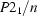

*a* = 11.0075 (3) Å
*b* = 10.5197 (3) Å
*c* = 15.4479 (4) Å
β = 93.967 (2)°
*V* = 1784.51 (8) Å^3^
*Z* = 4
Mo *K*α radiation
μ = 0.10 mm^−1^
*T* = 100 (2) K
0.20 × 0.15 × 0.10 mm

#### Data collection

Bruker SMART APEX diffractometer
Absorption correction: none
16316 measured reflections
4072 independent reflections
2646 reflections with *I* > 2σ(*I*)
*R*_int_ = 0.065

#### Refinement

*R*[*F* ^2^ > 2σ(*F* ^2^)] = 0.047
*wR*(*F* ^2^) = 0.120
*S* = 1.02
4072 reflections
255 parameters
2 restraints
H atoms treated by a mixture of independent and constrained refinement
Δρ_max_ = 0.26 e Å^−3^
Δρ_min_ = −0.26 e Å^−3^

### 

Data collection: *APEX2* (Bruker, 2007 ▶); cell refinement: *SAINT* (Bruker, 2007 ▶); data reduction: *SAINT*; program(s) used to solve structure: *SHELXS97* (Sheldrick, 2008 ▶); program(s) used to refine structure: *SHELXL97* (Sheldrick, 2008 ▶); molecular graphics: *X-SEED* (Barbour, 2001 ▶); software used to prepare material for publication: *publCIF* (Westrip, 2008 ▶).

## Supplementary Material

Crystal structure: contains datablocks global, I. DOI: 10.1107/S1600536808026846/tk2298sup1.cif

Structure factors: contains datablocks I. DOI: 10.1107/S1600536808026846/tk2298Isup2.hkl

Additional supplementary materials:  crystallographic information; 3D view; checkCIF report

## Figures and Tables

**Table 1:** Hydrogen-bond geometry (Å, °)

*D*—H⋯*A*	*D*—H	H⋯*A*	*D*⋯*A*	*D*—H⋯*A*
O1—H1*o*⋯O2	0.87 (1)	1.81 (2)	2.599 (2)	150 (3)
N1—H1*n*⋯O3^i^	0.85 (1)	2.01 (1)	2.812 (2)	157 (2)

Symmetry code: (i) 
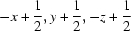
.

**Fig. 1.**
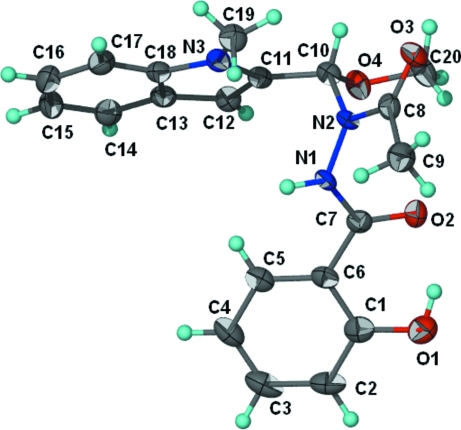
Thermal ellipsoid plot (Barbour, 2001) of (I) drawn at the 70% probability level. Hydrogen atoms are drawn as spheres of arbitrary radius.

## supplementary crystallographic information

### Comment

The Schiff base, *N*'-[(1-methyl-1*H*-indol-2-yl)methylene]-2-hydroxybenzohydrazide, exhibits useful medicinal properties (Jin *et al., *2006; Joshi *et al.*, 2008; Szczepankiewicz *et al., *2001). When dissolved in acetic anhydride, the compound undergoes a reaction to yield the title compound, (I), Fig. 1. Essentially, a mole of methyl acetate has been added across the carbon-nitrogen double-bond. In the crystal structure of (I), the amino group forms an N–H···O hydrogen bond to the acetyl group of an adjacent molecule to result in a zigzag chain that runs along the *b*-axis of the orthorhombic unit cell, Table 1. The 2-hydroxy group is internally hydrogen bonded to the amido group though an O–H···O hydrogen bond, Table 1.

### Experimental

2-Hydroxybenzohydrazide was condensed with 1-methylindole-3-carboxaldehyde to yield the corresponding Schiff base. To *N*'-[(1-methyl-1*H*-indol-2-yl)methylene]-2-hydroxybenzohydrazide (0.88 g, 3 mmol) was added acetic anhydride (10 ml). The mixture was heated to 398–403 K until the reactants dissolved completely. After 2 h of heating, the mixture was cooled and then treated with ethyl acetate and saturated aqueous sodium bicarbonate. The organic layer was separated and dried over anhydrous sodium sulfate. The solvent was evaporated and the resulting solid was recrystallized from methanol to give (I) as colorless crystals.

### Refinement

Carbon-bound H-atoms were placed in calculated positions (C—H 0.95 to 0.98 Å) and were included in the refinement in the riding model approximation, with *U*(H) set to 1.2–1.5*U*(C). The hydroxy- and ammonium H-atoms were located in a difference Fourier map, and were refined with a distance restraints O–H = N–H = 0.85±0.01 Å; their temperature factors were freely refined.

### Crystal data

**Table:**

C_20_H_21_N_3_O_4_	*F*_000_ = 776
*M**_r_* = 367.40	*D*_x_ = 1.367 Mg m^−^^3^
Monoclinic, *P*2_1_/*n*	Mo *K*α radiation λ = 0.71073 Å
Hall symbol: -P 2yn	Cell parameters from 1739 reflections
*a* = 11.0075 (3) Å	θ = 2.2–21.4º
*b* = 10.5197 (3) Å	µ = 0.10 mm^−^^1^
*c* = 15.4479 (4) Å	*T* = 100 (2) K
β = 93.967 (2)º	Block, colorless
*V* = 1784.51 (8) Å^3^	0.20 × 0.15 × 0.10 mm
*Z* = 4	

### Data collection

**Table:**

Bruker SMART APEX diffractometer	2646 reflections with *I* > 2σ(*I*)
Radiation source: fine-focus sealed tube	*R*_int_ = 0.065
Monochromator: graphite	θ_max_ = 27.5º
*T* = 100(2) K	θ_min_ = 2.2º
ω scans	*h* = −14→14
Absorption correction: None	*k* = −13→13
16316 measured reflections	*l* = −20→20
4072 independent reflections	

### Refinement

**Table:**

Refinement on *F*^2^	Secondary atom site location: difference Fourier map
Least-squares matrix: full	Hydrogen site location: inferred from neighbouring sites
*R*[*F*^2^ > 2σ(*F*^2^)] = 0.047	H atoms treated by a mixture of independent and constrained refinement
*wR*(*F*^2^) = 0.120	*w* = 1/[σ^2^(*F*_o_^2^) + (0.0466*P*)^2^ + 0.3679*P*] where *P* = (*F*_o_^2^ + 2*F*_c_^2^)/3
*S* = 1.02	(Δ/σ)_max_ = 0.001
4072 reflections	Δρ_max_ = 0.26 e Å^−^^3^
255 parameters	Δρ_min_ = −0.26 e Å^−^^3^
2 restraints	Extinction correction: none
Primary atom site location: structure-invariant direct methods	

### Special details

**Table:**

Geometry. All e.s.d.'s (except the e.s.d. in the dihedral angle between two l.s. planes) are estimated using the full covariance matrix. The cell e.s.d.'s are taken into account individually in the estimation of e.s.d.'s in distances, angles and torsion angles; correlations between e.s.d.'s in cell parameters are only used when they are defined by crystal symmetry. An approximate (isotropic) treatment of cell e.s.d.'s is used for estimating e.s.d.'s involving l.s. planes.

### Fractional atomic coordinates and isotropic or equivalent isotropic displacement parameters (Å^2^)

**Table:**

	*x*	*y*	*z*	*U*_iso_*/*U*_eq_	
O1	0.75833 (13)	0.62701 (15)	0.28349 (10)	0.0328 (4)	
H1O	0.717 (2)	0.5560 (16)	0.2811 (18)	0.069 (10)*	
O2	0.57533 (11)	0.47150 (13)	0.28757 (9)	0.0266 (3)	
O3	0.27682 (12)	0.25872 (13)	0.22300 (9)	0.0279 (3)	
O4	0.41144 (11)	0.35118 (13)	0.43051 (8)	0.0257 (3)	
N1	0.38731 (14)	0.55433 (15)	0.29735 (10)	0.0197 (4)	
H1N	0.3430 (17)	0.6189 (15)	0.3061 (14)	0.037 (6)*	
N2	0.33109 (13)	0.43653 (14)	0.29605 (10)	0.0188 (3)	
N3	0.12618 (13)	0.52539 (15)	0.40494 (10)	0.0205 (4)	
C1	0.68446 (17)	0.7144 (2)	0.31706 (12)	0.0262 (5)	
C2	0.73287 (19)	0.8337 (2)	0.33795 (14)	0.0315 (5)	
H2	0.8144	0.8530	0.3262	0.038*	
C3	0.6634 (2)	0.9230 (2)	0.37533 (14)	0.0354 (5)	
H3	0.6972	1.0042	0.3892	0.042*	
C4	0.54370 (19)	0.8971 (2)	0.39356 (13)	0.0308 (5)	
H4	0.4966	0.9592	0.4209	0.037*	
C5	0.49478 (18)	0.78036 (18)	0.37132 (12)	0.0249 (4)	
H5	0.4132	0.7621	0.3837	0.030*	
C6	0.56213 (17)	0.68846 (18)	0.33118 (12)	0.0225 (4)	
C7	0.51091 (17)	0.56257 (18)	0.30497 (11)	0.0207 (4)	
C8	0.31298 (16)	0.36884 (19)	0.22067 (12)	0.0221 (4)	
C9	0.33655 (19)	0.4356 (2)	0.13833 (13)	0.0294 (5)	
H9A	0.3008	0.3867	0.0889	0.044*	
H9B	0.4246	0.4435	0.1337	0.044*	
H9C	0.2997	0.5204	0.1383	0.044*	
C10	0.30451 (16)	0.38509 (18)	0.38166 (12)	0.0212 (4)	
H10	0.2504	0.3091	0.3736	0.025*	
C11	0.24263 (16)	0.48432 (18)	0.43178 (12)	0.0208 (4)	
C12	0.28551 (17)	0.55066 (18)	0.50327 (12)	0.0227 (4)	
H12	0.3624	0.5400	0.5343	0.027*	
C13	0.19310 (17)	0.63891 (18)	0.52267 (12)	0.0222 (4)	
C14	0.18300 (18)	0.73351 (19)	0.58605 (12)	0.0261 (5)	
H14	0.2474	0.7477	0.6291	0.031*	
C15	0.07752 (18)	0.8057 (2)	0.58468 (13)	0.0282 (5)	
H15	0.0704	0.8708	0.6267	0.034*	
C16	−0.01854 (18)	0.7841 (2)	0.52232 (13)	0.0295 (5)	
H16	−0.0899	0.8348	0.5230	0.035*	
C17	−0.01235 (17)	0.6909 (2)	0.45972 (13)	0.0260 (5)	
H17	−0.0785	0.6754	0.4182	0.031*	
C18	0.09490 (16)	0.62076 (18)	0.46006 (12)	0.0220 (4)	
C19	0.05096 (17)	0.4838 (2)	0.32897 (12)	0.0269 (5)	
H19A	−0.0351	0.4872	0.3415	0.040*	
H19B	0.0726	0.3963	0.3144	0.040*	
H19C	0.0647	0.5397	0.2799	0.040*	
C20	0.4677 (2)	0.24041 (19)	0.39780 (14)	0.0313 (5)	
H20A	0.5395	0.2183	0.4358	0.047*	
H20B	0.4924	0.2574	0.3392	0.047*	
H20C	0.4097	0.1696	0.3960	0.047*	

### Atomic displacement parameters (Å^2^)

**Table:**

	*U*^11^	*U*^22^	*U*^33^	*U*^12^	*U*^13^	*U*^23^
O1	0.0245 (7)	0.0335 (9)	0.0408 (9)	−0.0008 (7)	0.0048 (6)	0.0009 (7)
O2	0.0224 (7)	0.0240 (8)	0.0339 (8)	0.0036 (6)	0.0054 (6)	−0.0027 (6)
O3	0.0311 (8)	0.0184 (8)	0.0342 (8)	−0.0058 (6)	0.0016 (6)	−0.0067 (6)
O4	0.0282 (7)	0.0211 (8)	0.0272 (7)	0.0051 (6)	−0.0028 (6)	−0.0001 (6)
N1	0.0201 (8)	0.0107 (8)	0.0284 (9)	−0.0007 (7)	0.0024 (7)	−0.0012 (7)
N2	0.0219 (8)	0.0112 (8)	0.0235 (8)	−0.0031 (6)	0.0023 (6)	−0.0011 (7)
N3	0.0213 (8)	0.0190 (9)	0.0213 (8)	−0.0025 (7)	0.0010 (6)	−0.0028 (7)
C1	0.0270 (10)	0.0277 (12)	0.0236 (10)	−0.0010 (9)	−0.0011 (8)	0.0057 (9)
C2	0.0291 (11)	0.0302 (12)	0.0345 (12)	−0.0109 (10)	−0.0027 (9)	0.0081 (10)
C3	0.0452 (13)	0.0230 (12)	0.0364 (12)	−0.0101 (10)	−0.0088 (10)	0.0048 (10)
C4	0.0420 (13)	0.0197 (11)	0.0297 (11)	−0.0021 (9)	−0.0028 (10)	−0.0017 (9)
C5	0.0296 (10)	0.0209 (11)	0.0239 (10)	−0.0027 (9)	−0.0014 (8)	0.0013 (9)
C6	0.0252 (10)	0.0184 (10)	0.0236 (10)	−0.0025 (8)	0.0000 (8)	0.0049 (8)
C7	0.0243 (10)	0.0205 (10)	0.0172 (9)	−0.0013 (8)	0.0020 (7)	0.0030 (8)
C8	0.0194 (9)	0.0207 (11)	0.0259 (10)	0.0033 (8)	0.0002 (8)	−0.0026 (8)
C9	0.0338 (11)	0.0306 (12)	0.0241 (10)	−0.0007 (10)	0.0028 (9)	−0.0032 (9)
C10	0.0228 (9)	0.0176 (10)	0.0232 (10)	−0.0010 (8)	0.0007 (8)	0.0005 (8)
C11	0.0222 (9)	0.0175 (10)	0.0228 (9)	−0.0016 (8)	0.0022 (8)	0.0017 (8)
C12	0.0238 (10)	0.0231 (11)	0.0213 (9)	0.0003 (8)	0.0008 (8)	0.0007 (8)
C13	0.0251 (10)	0.0199 (10)	0.0218 (10)	−0.0024 (8)	0.0036 (8)	0.0030 (8)
C14	0.0304 (11)	0.0268 (12)	0.0216 (10)	−0.0033 (9)	0.0043 (8)	−0.0024 (9)
C15	0.0356 (11)	0.0238 (11)	0.0263 (10)	0.0006 (9)	0.0090 (9)	−0.0047 (9)
C16	0.0282 (11)	0.0289 (12)	0.0326 (11)	0.0042 (9)	0.0101 (9)	0.0009 (10)
C17	0.0229 (10)	0.0266 (11)	0.0286 (11)	−0.0011 (8)	0.0036 (8)	0.0012 (9)
C18	0.0236 (9)	0.0193 (10)	0.0236 (10)	−0.0040 (8)	0.0059 (8)	0.0000 (8)
C19	0.0247 (10)	0.0279 (12)	0.0274 (11)	−0.0023 (9)	−0.0028 (8)	−0.0050 (9)
C20	0.0367 (12)	0.0185 (11)	0.0378 (12)	0.0085 (9)	−0.0026 (10)	0.0007 (9)

### Geometric parameters (Å, °)

**Table:**

O1—C1	1.354 (2)	C8—C9	1.491 (3)
O1—H1O	0.871 (10)	C9—H9A	0.9800
O2—C7	1.233 (2)	C9—H9B	0.9800
O3—C8	1.226 (2)	C9—H9C	0.9800
O4—C10	1.400 (2)	C10—C11	1.492 (3)
O4—C20	1.428 (2)	C10—H10	1.0000
N1—C7	1.360 (2)	C11—C12	1.363 (3)
N1—N2	1.385 (2)	C12—C13	1.424 (3)
N1—H1N	0.852 (9)	C12—H12	0.9500
N2—C8	1.368 (2)	C13—C14	1.406 (3)
N2—C10	1.477 (2)	C13—C18	1.413 (3)
N3—C18	1.375 (2)	C14—C15	1.387 (3)
N3—C11	1.389 (2)	C14—H14	0.9500
N3—C19	1.456 (2)	C15—C16	1.399 (3)
C1—C2	1.393 (3)	C15—H15	0.9500
C1—C6	1.406 (3)	C16—C17	1.382 (3)
C2—C3	1.365 (3)	C16—H16	0.9500
C2—H2	0.9500	C17—C18	1.392 (3)
C3—C4	1.393 (3)	C17—H17	0.9500
C3—H3	0.9500	C19—H19A	0.9800
C4—C5	1.375 (3)	C19—H19B	0.9800
C4—H4	0.9500	C19—H19C	0.9800
C5—C6	1.390 (3)	C20—H20A	0.9800
C5—H5	0.9500	C20—H20B	0.9800
C6—C7	1.485 (3)	C20—H20C	0.9800
			
C1—O1—H1O	106.0 (19)	O4—C10—C11	107.19 (14)
C10—O4—C20	112.71 (14)	N2—C10—C11	109.52 (15)
C7—N1—N2	120.11 (16)	O4—C10—H10	109.6
C7—N1—H1N	121.0 (15)	N2—C10—H10	109.6
N2—N1—H1N	117.1 (15)	C11—C10—H10	109.6
C8—N2—N1	121.13 (15)	C12—C11—N3	110.09 (17)
C8—N2—C10	123.05 (15)	C12—C11—C10	129.33 (17)
N1—N2—C10	115.52 (14)	N3—C11—C10	120.52 (16)
C18—N3—C11	107.92 (15)	C11—C12—C13	106.97 (16)
C18—N3—C19	124.45 (16)	C11—C12—H12	126.5
C11—N3—C19	127.50 (16)	C13—C12—H12	126.5
O1—C1—C2	118.06 (18)	C14—C13—C18	118.61 (18)
O1—C1—C6	122.32 (18)	C14—C13—C12	134.43 (18)
C2—C1—C6	119.6 (2)	C18—C13—C12	106.95 (17)
C3—C2—C1	120.1 (2)	C15—C14—C13	118.93 (18)
C3—C2—H2	119.9	C15—C14—H14	120.5
C1—C2—H2	119.9	C13—C14—H14	120.5
C2—C3—C4	121.1 (2)	C14—C15—C16	120.91 (19)
C2—C3—H3	119.4	C14—C15—H15	119.5
C4—C3—H3	119.4	C16—C15—H15	119.5
C5—C4—C3	118.9 (2)	C17—C16—C15	121.71 (19)
C5—C4—H4	120.5	C17—C16—H16	119.1
C3—C4—H4	120.5	C15—C16—H16	119.1
C4—C5—C6	121.40 (19)	C16—C17—C18	117.16 (18)
C4—C5—H5	119.3	C16—C17—H17	121.4
C6—C5—H5	119.3	C18—C17—H17	121.4
C5—C6—C1	118.71 (18)	N3—C18—C17	129.29 (17)
C5—C6—C7	122.54 (17)	N3—C18—C13	108.07 (16)
C1—C6—C7	118.73 (18)	C17—C18—C13	122.64 (18)
O2—C7—N1	121.25 (17)	N3—C19—H19A	109.5
O2—C7—C6	122.64 (17)	N3—C19—H19B	109.5
N1—C7—C6	116.05 (17)	H19A—C19—H19B	109.5
O3—C8—N2	119.69 (18)	N3—C19—H19C	109.5
O3—C8—C9	123.09 (18)	H19A—C19—H19C	109.5
N2—C8—C9	117.21 (17)	H19B—C19—H19C	109.5
C8—C9—H9A	109.5	O4—C20—H20A	109.5
C8—C9—H9B	109.5	O4—C20—H20B	109.5
H9A—C9—H9B	109.5	H20A—C20—H20B	109.5
C8—C9—H9C	109.5	O4—C20—H20C	109.5
H9A—C9—H9C	109.5	H20A—C20—H20C	109.5
H9B—C9—H9C	109.5	H20B—C20—H20C	109.5
O4—C10—N2	111.40 (15)		
			
C7—N1—N2—C8	−84.8 (2)	N1—N2—C10—C11	48.99 (19)
C7—N1—N2—C10	89.2 (2)	C18—N3—C11—C12	0.6 (2)
O1—C1—C2—C3	−177.23 (18)	C19—N3—C11—C12	176.65 (18)
C6—C1—C2—C3	2.8 (3)	C18—N3—C11—C10	−176.91 (16)
C1—C2—C3—C4	0.2 (3)	C19—N3—C11—C10	−0.9 (3)
C2—C3—C4—C5	−1.6 (3)	O4—C10—C11—C12	11.2 (3)
C3—C4—C5—C6	−0.1 (3)	N2—C10—C11—C12	−109.8 (2)
C4—C5—C6—C1	3.0 (3)	O4—C10—C11—N3	−171.84 (16)
C4—C5—C6—C7	−178.81 (18)	N2—C10—C11—N3	67.2 (2)
O1—C1—C6—C5	175.68 (17)	N3—C11—C12—C13	−0.6 (2)
C2—C1—C6—C5	−4.3 (3)	C10—C11—C12—C13	176.68 (18)
O1—C1—C6—C7	−2.6 (3)	C11—C12—C13—C14	−178.7 (2)
C2—C1—C6—C7	177.42 (17)	C11—C12—C13—C18	0.3 (2)
N2—N1—C7—O2	19.8 (3)	C18—C13—C14—C15	−0.2 (3)
N2—N1—C7—C6	−162.92 (15)	C12—C13—C14—C15	178.6 (2)
C5—C6—C7—O2	−163.41 (18)	C13—C14—C15—C16	1.0 (3)
C1—C6—C7—O2	14.8 (3)	C14—C15—C16—C17	−0.2 (3)
C5—C6—C7—N1	19.4 (3)	C15—C16—C17—C18	−1.3 (3)
C1—C6—C7—N1	−162.41 (17)	C11—N3—C18—C17	−179.76 (19)
N1—N2—C8—O3	171.07 (16)	C19—N3—C18—C17	4.0 (3)
C10—N2—C8—O3	−2.5 (3)	C11—N3—C18—C13	−0.4 (2)
N1—N2—C8—C9	−10.1 (2)	C19—N3—C18—C13	−176.59 (17)
C10—N2—C8—C9	176.32 (16)	C16—C17—C18—N3	−178.60 (19)
C20—O4—C10—N2	−71.2 (2)	C16—C17—C18—C13	2.1 (3)
C20—O4—C10—C11	168.97 (15)	C14—C13—C18—N3	179.21 (16)
C8—N2—C10—O4	104.46 (19)	C12—C13—C18—N3	0.1 (2)
N1—N2—C10—O4	−69.42 (19)	C14—C13—C18—C17	−1.4 (3)
C8—N2—C10—C11	−137.13 (17)	C12—C13—C18—C17	179.47 (18)

### Hydrogen-bond geometry (Å, °)

**Table:**

*D*—H···*A*	*D*—H	H···*A*	*D*···*A*	*D*—H···*A*
O1—H1*o*···O2	0.87 (1)	1.81 (2)	2.599 (2)	150 (3)
N1—H1*n*···O3^i^	0.85 (1)	2.01 (1)	2.812 (2)	157 (2)

Symmetry codes: (i) −*x*+1/2, *y*+1/2, −*z*+1/2.
